# The Effect of 8 Weeks Aerobic Exercise on Insulin Resistance in Type 2 Diabetes: A Randomized Clinical Trial

**DOI:** 10.5539/gjhs.v7n1p115

**Published:** 2014-08-15

**Authors:** Narges Motahari-Tabari, Marjan Ahmad Shirvani, Mahbobeh Shirzad-e-Ahoodashty, Elham Yousefi-Abdolmaleki, Mojgan Teimourzadeh

**Affiliations:** 1 School of Nursing & Midwifery, Mazandaran University of Medical Sciences, Sari, Iran; 2 Faculty of Medicine, Mazandaran University of Medical Sciences, Sari, Iran

**Keywords:** aerobic exercise, blood glucose, insulin resistance, physical activity, Type 2 diabetes

## Abstract

Diabetes complications are the main reasons behind morbidity and mortality preventable by healthy diet and physical activity. There are few studies about the effect of aerobic exercises on insulin resistance in human. Also various training protocols are associated with different results. Since approaches to decrease insulin resistance may be followed by more effectiveness treatment, this study assessed the effect of aerobic exercise on insulin resistance in Type 2 Diabetes Mellitus. In this randomized clinical trial, 53 Type 2 diabetic women were randomly divided into two groups as exercise (n=27) and control (n=26). The exercise protocol included warm-up by stretching and flexibility exercises for 10 m, followed by walking for 30 m with maximum intensity 60% increase in heart rate and then stretching in the seated position for 10 m, 3 times a week for 8 weeks. Resistance to insulin was assessed using Homeostasis Model Assessment of Insulin Resistance (HOMA-IR). Significant differences have been observed in insulin resistance, fasting glucose and plasma insulin between the groups after 8 weeks. There were significant differences in waist and hip circumference, BMI, plasma insulin and insulin resistance within the groups over time. In addition, the changes in waist and hip circumference, FBS, plasma insulin and insulin resistance had significant interaction with the time between the groups. The current exercise protocol has been effective in lowering plasma glucose (p = 0.05), insulin levels (p = 0.000) and insulin resistance (p = 0.02). It seems that aerobic exercises training promote the effectiveness of medical treatment in Type 2 Diabetes Mellitus.

## 1. Introduction

Diabetes mellitus is a syndrome of impaired carbohydrate metabolism, lipids and proteins caused by lack of insulin secretion or the tissues decreased sensitivity to insulin metabolic effects. The decreased insulin sensitivity is called Type 2 Diabetes Mellitus (T_2_ DM) or non-insulin dependent diabetes mellitus often referred to as insulin resistance (Guyton & Hall, 2010). Diabetes is one of the most important health problems and chronic metabolic diseases requiring critical care (Snowling & Hopkins, 2006). The prevalence of T_2_ DM has grown rapidly worldwide. According to the International Diabetes Federation, 246 million people are currently affected by diabetes and it is estimated to get to 380 million people by 2025 so that unhealthy diet and less physical activity seem to be the predisposing causes behind it (Teixeira-Lemos, Nunes, Teixeira & Reis, 2011; Tuomilehto et al., 2001). Diabetes side effects are the main causes of morbidity and mortality. Its short-term side effects can be prevented by taking medication accurately, following healthy diet and physical activity, thus the long-term complications will be delayed (Bate & Jerums, 2003).

Recent epidemiologic studies indicate that people with active physical life are less likely to develop impaired glucoses tolerance and non-insulin dependent diabetes (Conn et al., 2007; Katzmarzyk et al., 2003). Physical activity has been stated as one of the most important factors in the treatment of T_2_ DM (Praet & Van loon, 2007). Regular body exercise related effects on the decrease of insulin resistance and non-insulin dependent diabetes have been emphasized in several studies (Praet & van Loon, 2009; Teixeira-Lemos et al., 2011; Turcotte & Fisher, 2008). Researchers have discovered that the elderly with regular physical activity have higher glucose tolerance and less insulin response to glucose disorder compared to the sedentary individuals at the same age and weight (Ivy, 1997). There are convincing evidence that aerobic exercise can reduce the risk of glucose intolerance disorder in non-insulin dependent diabetes (Church, 2011; Praet & van Loon, 2009; Tuomilehto et al., 2001). However, some studies have concluded that evidence related to the effect of physical activity on the treatment of non-insulin dependent diabetes are not robust enough (Carroll & Dudfield, 2004; Ivy, 1997). Reviewing some of the early studies also did not reveal the effects of physical activity on non-insulin dependent diabetes, fasting blood glucose, insulin level and glucose tolerance (Ivy, 1997). Most of the recent studies have shown better results obtained through using long-term physical activity programs (Carroll & Dudfield, 2004; Praet & van Loon, 2009; Tuomilehto et al., 2001).

It seems that the type and duration of exercise affect the results. On the other hand, in most of the studies, the effect of exercise on insulin resistance hasn’t been assessed enough. Since to find out the approaches for insulin resistance decrease will be followed by more effectiveness of the medical treatments, delayed side effects and improved metabolic control of diabetes, the current study has assessed an eight week aerobic exercise protocol influencing insulin resistance in T_2_ DM.

## 2. Method

The current study is a randomized clinical trial approved by ethical committee of Mazandran Medical Science University. Fifty-three T_2_ DM female patients referring to two diabetes clinics in the North of Iran were divided into 2 groups of control and exercise by random allocation method. Groups were matched by age (>40 and <40) and Body Mass Index (BMI) (≥25 and <25). Four blocks were made for each group and random allocation was provided with coded cards. All of the eligible participants have read the information and signed the consent form. Sample size was calculated 25 patients for each group based on mean insulin plasma before (6.2±0.6) and after intervention (5.8±0.4), the coefficient interval 95%, power 90% and 10% dropout (Oberbach et al., 2006).

According to the America Diabetes Association, diagnostic criteria for T_2_ DM were defined this way the fasting plasma glucose concentration more than 126 mg/dl (7mmol/L) or plasma glucose concentration, 2 hours after meal, 200 mg/dl or higher (11.1 mmol/L) in 2 different times (American Diabetes Association, 2004). Inclusion criteria were 30-65 years old, housewife, females from north of Iran, with T_2_ DM, agreeing to participate in the study and receiving only oral medicine. Exclusion criteria included acute and chronic inflammatory diseases, heart diseases, pregnancy, smoking, alcohol and drug addiction, muscular disorders and patients who often do exercises (Oberbach et al., 2006). Patients were examined by the clinic doctors for heart or cardiovascular diseases incompatible with the physical activity. All the patients were weighed shoeless and wearing light clothes by the same scale and their height was measured using wall meter. In addition, a standard fabric meter was used to measure the waist to hip ratio.

Demographic information was recorded and primary tests including fasting blood sugar (FBS) and fasting serum insulin were conducted. FBS was measured by Glucose kit (GOD, Pars Azmon product, made in Iran) with enzymatic and Colorimetric method (GOD-PAP) and photometric methods for single point measurement. FBS was measured based on mmol/L through formula [Glucose mmol/L = 0.05551 × Glucose (mg/dl)]. Serum Insulin were measured by insulin kit (300-2475: product cod, made in the USA) with enzyme immunoassay colorimetric method, where the normal level for Type 2 diabetes was considered 0.7-25 µ/IU/ ml. Insulin resistance was measured using HOMA- IR formula. It is a method used to quantify insulin resistance and beta-cell function.


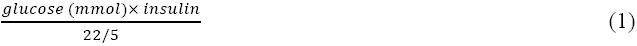


Based on the homeostasis model assessment of insulin resistance criteria (HOMA-IR), the levels lower than 2.24 were considered as insulin sensitivity as and higher than these as insulin resistant (Matthews et al., 1985).

Every four week, the anthropometric measurements and blood tests were repeated. All of the tests were performed in the same laboratory by the same person using the same kit. The participants’ calorie intake was controlled via the nutrition form provided by Diabetic Control Center. In the intervention group, the exercise protocol was applied based on the study by Oberbach et al. (2006) and Carroll and Dudfield (2004). In this manner that 50 minute daily moderate to intense aerobic exercise was performed 3 times a week for 8 weeks being supervised by two trainers and the researcher. This activity included 10 minute exercise movements in standing position to warm up. Following with 30 minute brisk walk where the activity intensity was measured by 60% maximum heart rate for each individual (Nieman, 1999), using:


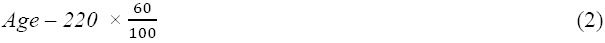


The researcher controlled heart beat 2 times through carotid pulse. At the end, in order to restore the body to its initial state and cooling, 10 minute exercise movements were performed in the seated position. In addition, all of the patients could drink water during their training. All of the measurements were performed in the control group with no intervention.

Descriptive statistics (mean, frequency), chi-square, Fisher’s Exact Test, Independent T-test and repeated measurement were used to analyze data by SPSS version 16.

## 3. Results

Sixty-two patients were included in this study, 4 of whom were excluded due to the exclusion criteria, and then 5 patients were exclude because of their irregular attendance in the program and lost to follow up program during the first month. At the end, 27 patients in the exercise and 26 in the control group were assessed. The mean age was 49.29 (SE: 1.12, median: 50, rang: 27) and 49.0 (SE: 1.60, median: 49, rang: 32) in the exercise and control groups, respectively, which had no significance difference. Most of the patients in both groups had primary level education (48.1% exercise group, 42.3% control group). There was no significant difference between the groups in terms of the education level. All the patients in both groups were housewives. Comparing the anthropometric measurements and blood parameters between the groups did not reveal any significant differences at the onset of the study and during the first month later, but FBS and plasma insulin had decreased significantly in the exercise group after 8 weeks (Table 1, 2, 3).

**Table 1:** Anthropometric and laboratory parameters of control and exercise group in the onset of study

Parameter	Exercise group		Control group				95% CI of the difference	P value

	Mean	SE	Rang	Mean	SE	Rang		
Weight(Kg)	72.66	2.55	53	73.92	2.12	43	-7.95 – 5.44	0.70
BMI(kg/m^2^)	29.74	0.85	18.68	30.35	0.89	18.23	-3.10 – 1.87	0.62
Waist circumference (cm)	93.42	1.60	37	94.76	1.72	32	-6.05 – 3.40	0.57
Hip circumference (cm)	107.22	1.75	41	107.65	1.70	31	-5.35 – 4.49	0.86
Waist/Hip(cm)	0.87	0.00	0.14	0.88	0.00	0.20	-0.03 – 0.01	0.45
FBS (mg/dl)	157.33	12.89	278	159.88	11.56	235	-37.41 –32.31	0.88
Plasma Insulin (μiu/ml)	6.15	0.92	17.90	6.73	0.83	16.60	-3.09 – 1.92	0.64

SE: standard error, CI: coefficient interval.

**Table 2:** Anthropometric and laboratory parameters of control and exercise group in the 4th week of the study

Parameter	Exercise group			Control group			95% CI of the difference	P value

	Mean	SE	Rang	Mean	SE	Rang		
Weight(Kg)	72.03	2.69	57	73.42	2.17	43	-8.36 – 5.59	0.69
BMI(kg/m^2^)	29.48	0.91	19.67	30.14	0.90	18.23	-3.25 – 1.93	0.60
Waist circumference (cm)	94.03	2.20	48	93.38	1.89	32	-5.19 – 6.49	0.82
Hip circumference (cm)	105.93	2.11	51	106.69	1.93	31	-6.53 – 5.00	0.79
Waist/Hip(cm)	0.88	0.00	0.16	0.87	0.00	0.20	-0.00 – 0.03	0.26
FBS (mg/dl)	157.96	10.24	203	157.58	9.90	235	-28.25 –29.02	0.97
Plasma Insulin (μiu/ml)	5.36	0.78	14	6.18	0.63	16.60	-2.58 – 1.20	0.41

SE: standard error, CI: coefficient interval.

**Table 3:** Anthropometric and laboratory parameters of control and exercise group in the 8th week of the study

Parameter	Exercise group		Control group				95% CI of the difference	P value

	Mean	SE	Rang	Mean	SE	Rang		
Weight(Kg)	71.42	2.67	58	74.04	2.23	45	-9.68 – 4.45	0.69
BMI(kg/m^2^)	29.23	0.91	20.45	30.31	0.94	16.77	-3.72 – 1.55	0.60
Waist circumference (cm)	89.85	1.84	44	94	1.68	31	-9.18 – 0.88	0.82
Hip circumference (cm)	101.19	2.30	58	106.52	1.92	33	-11.42 – 0.75	0.79
Waist/Hip(cm)	0.89	0.00	0.18	0.88	0.00	0.26	-0.02 – 0.03	0.26
FBS (mg/dl)	134.85	7.91	153	162.76	1.67	282	-55.86 –0.05	0.97
Plasma Insulin (μiu/ml)	3.62	0.42	8.90	6.43	0.50	13	-4.12 – 1.50	0.41

SE: standard error, CI: coefficient interval

According to the repeated measurements, there were significant differences in weight (p=0.01), waist circumference (p=0.004), hip circumference (p=0.000), BMI (p=0.01), plasma insulin (p=0.002), and insulin resistance (p=0.004) within the groups over time. In addition, changes in waist circumference (p=0.004), hip circumference (p=0.000), FBS (p=0.06), plasma insulin (p=0.007) and insulin resistance (p=0.007) had significant interaction with the time between the groups.

There was no meaningful difference in insulin resistance between the groups at the beginning of the study and after the first and second month, but it was significantly lower in the exercise group after 2 months (Table 4).

**Table 4:** Comparison of insulin resistance in the exercise and control group during the study

Insulin Resistance	Exercise group N (%)	control group N (%)	P-value
Onset of the study			
resistant	9 (33.3)	11 (42.3)	0.34
non-resistance	18 (66.7)	15 (57.7)	
After one month			
resistant	8 (29.6)	12 (46.2)	0.68
non-resistance	19 (70.4)	14 (53.8)	
After Second month			
resistant	3 (11.1)	10 (38.5)	0.02
non -resistance	24 (88.9)	16 (61.5)	

The amount of drug intake did not show a meaningful difference during the study. The mean consumed pills at the onset of the study was 4.33±1.64 in the exercise and 4.57±2.21 in the control group, that after one month, it changed to 4.55±2.02 and 4.61±2.45 and after two months, it changed to 4.66±2.14 and 5.08±2.32 in the exercise and control group, respectively.

## 4. Discussion

Several studies have assessed the effects of physical activity on the improvement of diabetes’ different aspects. Various exercise protocols have been employed in these studies and various results have been reported (Boule, Kenny, Haddade, Wells, & Sigal, 2003; Church, 2011; Ivy, 1997; Praet & van Loon, 2009). Since muscle contraction increases glucose uptake in skeletal muscles, physical activity has been suggested in T2 DM (Praet & van Loon, 2009; Teixeira-Lemos et al., 2011). In the current study, moderate to intense levels of aerobic exercises were performed since it affects a large group of muscles over time. There were significant differences in fasting glucose, plasma insulin and insulin resistance in the exercise group compared with the control group following 8 weeks. It seems that physical activity has a meaningful relationship with the insulin effectiveness increase in the skeletal muscles (Guyton & Hall, 2010; Ivy, 1997; Teixeira-Lemos et al., 2011; Turcotte & Fisher, 2008). This effect is related to the promotion of glucose uptake in the skeletal muscles, loss of body fat in the body central part (Guyton & Hall, 2010; Praet & van Loon, 2009; Teixeira-Lemos et al., 2011; Turcotte & Fisher, 2008), the lipid products reduction and lipid oxidative capacity increase in the muscle cells (Turcotte & Fisher, 2008), the insulin function increase in the organs cells involved in the exercise, the positive regulation of signaling pathway stimulation by insulin (Teixeira-Lemos et al., 2011) glycogen reserve decrease in liver and muscles (Praet & van Loon, 2009), the inflammatory markers change (Oberbach et al., 2006; Praet & van Loon, 2009), the prevention of muscle atrophy, new muscle tissue being built and capillary network congestion increase in muscles (Guyton & Hall, 2010; Praet & van Loon, 2009; Teixeira-Lemos et al., 2011).

Considering the study results, it seems that the effect of the exercise depends on various factors such as the type, intensity and frequency of training and glucose levels. In this study, the positive effect of aerobic exercise on glucose and insulin level appeared after 8 weeks. Some other studies reported the impact of prolong exercise protocols on insulin level improvement (Kirwan et al., 2009; Kodama et al., 2007; Teixeira-Lemos et al., 2011). Some investigators reported the effect of endurance and resistance training (Fenicchia et al., 2004; Praet & van Loon, 2009; Tuomilehto et al., 2001). On the other hand, some research cases have suggested aerobic and resistance exercise together (Boule et al., 2003; Church, 2011; Kodama et al., 2007) Oberbach et al. (2006) added swimming to aerobic training protocol and reported improvement in glucose metabolism and insulin resistance after 4 week training. Although the aerobic protocol in the study of Oberbach et al. (2006) was near to that obtained by us, the earlier effect may be due to adding resistance exercise. Kodama et al. (2007) reported combining aerobic and endurance exercise even with low intensity and volume could improve insulin resistance. Also Church (2011) said the combination of aerobic and resistance exercise is more effective than on their own.

The intensity of exercise is also an important factor. Some studies denoted that exercise in terms of intensity increases sensitivity to insulin (Hayashi et al., 2005; Rhéaume et al., 2003). In this study, moderate to intense exercise was performed (60% vo2max). Most of the studies suggested the protocols with intensity of more than 50% vo2max (Hayashi et al., 2005; Kirwan et al., 2009). Since exercise strength depends on its energy consumption effects, if the intensity of exercise is not sufficient, the duration must be increased, so that the energy consumption could change glucose homeostasis (Praet & Van loon, 2007).

Some authors showed that glucose initial level influenced the results, as there was low response to exercise in the healthy people (Fenicchia et al., 2004; Oberbach et al., 2006). We observed the positive effect of exercising in nearly 157 mg/dl level for FBS and 6 μiu/ml for plasma insulin.

Some others reported the weight change could be an important influential factor on the exercise outcome (Carroll & Dudfield, 2004; Li et al., 2008; Lindstrom et al., 2006; Praet & Van loon, 2007). It seems that insulin resistance is accompanied by increased abdominal fat and reduced muscle mass, thus an exercise program focusing on the central body fat loss could have effect on insulin resistance (Ivy, 1997). In the current study, although weight loss was significant in the exercise group, the difference between the groups was not meaningful. However, insulin resistance has improved. There are other reports about diabetes improvement by exercise without noticeable weight loss. According to Tuomilehto et al. (2001), exercise for more than 4 hours on a weekly basis could reduce the risk of diabetes without weight loss. Church (2011) said even relatively low weight loss can reduce the risk of Type 2 diabetes. Teixeira-Lemos et al. (2011) reviewed the effect of regular exercise and concluded that total, visceral and subcutaneous fat decrease occurs following the regular exercise and improved diabetes via glycaemia control and increase of free fatty acids oxidation without weight loss. However, according to Oberbach et al. (2006), the decrease in BMI may be seen by the combination of endurance exercise and aerobic training. Some researchers reported that in addition to changing Type 2 diabetic patients’ life style, exercise has positive effects on weight loss, waist circumference, fasting glucose plasma and insulin serum levels (Carroll & Dudfield, 2004; Church, 2011; Tuomilehto et al., 2001). On the other hand, increasing insulin sensitivity has a significant correlation with changes in body fat and anti-inflammatory factors (Bluher et al., 2005; Engeli et al., 2003; Nicklas et al., 2004; Praet & van Loon, 2009; Weyer et al., 2010). Thus, change in life style in addition to training protocol may be result to more weight loss and so earlier improvement in glycaemia and insulin resistance.

There were some limitations in the current study. The immediate effect of exercise on glucose and insulin levels after training and change in anti inflammatory factors during the study were not assessed, which are suggested for further studies.

## 5. Conclusion

The eight week moderate to intensive aerobic exercise protocol, 3 times a week was effective on insulin resistance in T2 DM patients. Several factors could affect the exercise outcome on glucose and insulin plasma levels and insulin resistance in T2 DM. Sufficient exercise program is one of the most important factors influencing the results in addition to population differences, life style and initial amounts of laboratory parameters. Therefore, it seems that in order to acquire the desired outcome, all of the above factors must be taken into account.
